# A nomogram involving immune‐inflammation index for predicting distant metastasis‐free survival of major salivary gland carcinoma following postoperative radiotherapy

**DOI:** 10.1002/cam4.5167

**Published:** 2022-09-01

**Authors:** Wenbin Yan, Xiaomin Ou, Chunying Shen, Chaosu Hu

**Affiliations:** ^1^ Department of Radiation Oncology Fudan University Shanghai Cancer Center Shanghai China; ^2^ Department of Oncology Shanghai Medical College Shanghai China

**Keywords:** distant metastasis, nomogram, postoperative radiotherapy, salivary gland carcinoma

## Abstract

**Background:**

Postoperative radiotherapy (PORT) is beneficial in the improvement of local‐regional control and overall survival (OS) for major salivary gland carcinomas (SGCs), and distant metastasis remained the main failure pattern. This study was designed to develop a nomogram model involving immune‐inflammation index to predict distant metastasis‐free survival (DMFS) of major SGCs.

**Patients and Methods:**

A total of 418 patients with major SGCs following PORT were randomly divided into a training (*n* = 334) and validation set (*n* = 84). The pre‐radiotherapy neutrophil‐to‐lymphocyte ratio (NLR), and platelet‐to‐lymphocyte ratio (PLR) were calculated and transformed as continuous variables for every patient. Associations between DMFS and variables were performed by univariate and multivariable analysis using Log‐rank and Cox regression methods. A nomogram was constructed based on the prognostic factors identified by the Cox hazards model. The decision curve analysis (DCA) was conducted with the training and validation set.

**Results:**

The estimated 3‐, 5‐, and 10‐year DMFS were 79.4%, 71.8%, and 59.1%, respectively. The multivariate analysis revealed that age (*p* = 0.033), advanced T stage (*p* = 0.003), positive *N* stage (*p* < 0.001), high‐risk pathology (*p* = 0.011), and high PLR (*p* = 0.001) were significantly associated with worse DMFS. The nomogram showed good calibration and discrimination in the training (AUC = 80.9) and validation set (AUC = 87.9). Furthermore, the DCA demonstrated favorable applicability, and a significant difference (*p* < 0.001) was observed for the DMFS between the subgroups based on the nomogram points.

**Conclusion:**

The nomogram incorporating clinicopathological features and PLR presented accurate individual prediction for DMFS of the patients with major SGCs following PORT. Further external validation of the model is warranted for clinical utility.

## Graphical abstract

The nomogram predicting distant‐metastasis‐free survival(DMFS) based on the clinicopathological factors and immune‐inflammation index for the major salivary gland carcinoma.

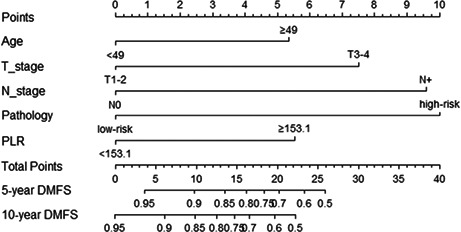

## 1 INTRODUCTION

The cancers of major salivary gland (SGCs) are rare malignant tumors accounting for <1% of all cancers. Surgical resection of the primary tumor is the major treatment for the non‐metastatic SGCs.^1^ Additionally, postoperative radiotherapy (PORT) was strongly recommended for those patients with adverse factors such as high grade, advanced stage, and positive lymph nodes owing to better local‐control rate and overall survival (OS).^2^, ^3^ A favorable prognosis was achieved for the patients following PORT with the estimated 5‐, and 10‐year OS being 63.9%–78% and 50%–75%, respectively.^4^, ^5^ However, distant metastasis remained the most common failure pattern.

The SGCs was composed of a variety of pathological subtypes, and high‐grade histology was associated with high risk of distant metastasis.^6^ Moreover, several clinicopathological characteristics including advanced T stage, positive nodal involvement,^7^ lymphovascular invasion (LVI)^8^ were significantly correlated with distant metastasis‐free survival (DMFS) for the patients who underwent PORT.

The tumor microenvironment was important in the development and response to treatment for various cancers. Of which, the systemic immune‐inflammation biomarkers were significant prognostic predictors for a variety of solid tumors,^9^, ^10^ and counts could be easily accessed by clinical practice. Several studies have indicated that the pre‐treatment platelet‐to‐lymphocyte ratio (PLR) and neutrophil‐to‐lymphocyte ratio (NLR) were independent predictors for survival in various cancers.^11^, ^12^, ^13^ Moreover, the elevated PLR and NLR were correlated with poor OS in the head and neck cancer (HNCC).^14^ Nevertheless, the histology of SGCs was different from the squamous cancer of HNCC, and associations between PLR, NLR and DMFS were inclusive in the SGCs.

Thus, we conducted this study to investigate the prognostic value of PLR and NLR for DMFS in the SGCs, and the nomogram was established to accurately predict treatment outcomes in SGCs. The identification of patients with poor DMFS could be helpful in the selection of cases whose outcome may be improved with adjuvant therapy.

## 2 METHODS

### 2.1 Patients and study design

A retrospective study was conducted on a primary cohort of patients diagnosed with salivary gland carcinoma who underwent surgery and radiotherapy in Fudan Cancer Shanghai Cancer Center (Shanghai, China) between November 2004 and November 2020. Inclusion criteria were as follows: histopathological confirmation as malignancy of major salivary glands; surgical resection of primary tumor site; adjuvant radiotherapy delivered with curative intent. Specific exclusion criteria were as follows: distant metastasis at initial diagnosis, with no treatment of surgery, incomplete radiotherapy, treatment data and follow‐up information unavailable.

The study was designed by guidelines of the TRIPOD (Transparent Reporting of a multivariable prediction model for Individual Prognosis or Diagnosis) Statement Checklist.^15^ As the data of participants were collected retrospectively through medical records, ethical approval was obtained through the ethics committee from Fudan University Shanghai Cancer Center (SCCIRB) (2011227–11).

### 2.2 Treatment

The decisions of treatment were consistent with the discussion of the head and neck oncology multidisciplinary board. All the patients underwent resection of primary tumor site, and adjuvant radiotherapy was performed for the patients with adverse features as: advanced stage, high‐grade histology, perineural invasion (PNI), LVI, and positive surgical margin. Radiotherapy was delivered using intensity‐modulated radiation therapy or 3D‐CRT once daily five times per week. Chemotherapy was rarely used after surgery.

### 2.3 Endpoints of the study

The distant metastasis was detected by the follow‐up imaging including chest CT, ECT, or PET‐CT. The primary endpoint was distant metastasis‐free survival (DMFS) which was calculated from the date of initial diagnosis to the date of distant metastasis, or death of any cause, or last follow‐up. OS was defined as the duration between date of initial diagnosis and the date of death of any cause, or last follow‐up.

### 2.4 Selection of the variables

**FIGURE 1:**
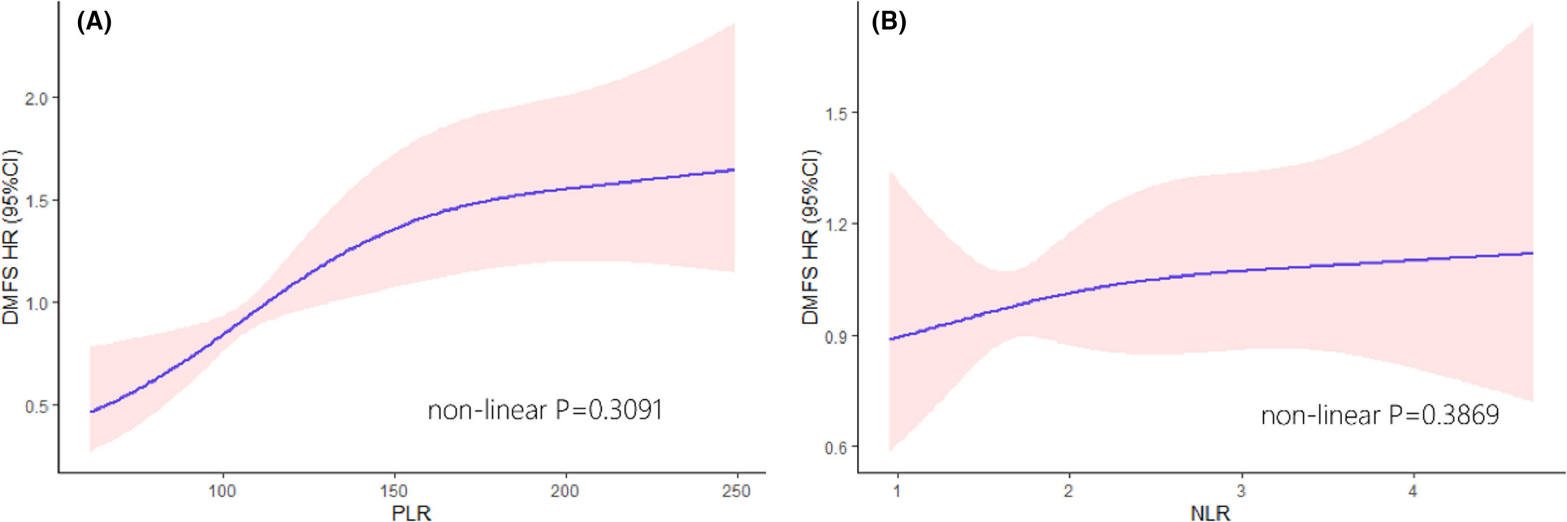
Association of the estimated DMFS HR (95% CI) and PLR (A), NLR (B)

The peripheral biomarkers including platelets, lymphocyte count, and neutrophil count were obtained from blood routine tests before PORT. The platelet–lymphocyte ratio (PLR) was calculated as the absolute platelet count measured in 10^9 divided by the absolute lymphocyte count measured in 10^9. Similarly, the neutrophil‐lymphocyte ratio (NLR) represented the ratio of neutrophil count to lymphocyte count. The continuous variables (age, PLR, NLR) were evaluated using restricted cubic splines,^16^ and transferred as categorical factors by maximally selected rank statistics method^17^ (Figure 1). The clinicopathological features were enrolled based on previous reports. The high‐risk pathology included: adenoid cystic carcinoma, salivary duct carcinoma, carcinosarcoma, undifferentiated carcinoma, adenocarcinoma, high‐grade mucoepidermoid carcinoma, high‐grade carcinoma ex‐pleomorphic adenoma, and lymphoepithelial carcinoma.^18^

### 2.5 Development and validation of the model

The cohort was randomly divided into a training and validation cohort (8:2). A Cox proportional hazards regression was conducted to identify the independent predictors in the training set. The final model was determined with a backward step‐down process using the Akaike information criterion as the stopping rule.^16^ The concordance index (C‐index) which compares the survival probability predicted by the nomogram versus that estimated by Kaplan–Meier was used to access the predictive value of the model. Calibration was assessed using 1000 bootstrap resamples^19^ in the training and validation cohort. The sensitivity and specificity of the nomogram were assessed by receiver operating characteristic (ROC) curves and the area under the curve (AUC) value. Furthermore, decision curve analysis (DCA) was performed for the evaluation of clinical utility.

### 2.6 Statistical analysis

All the characteristics were descriptively summarized using means and standard deviations for continuous variables. Categorical variables were listed upon clinical findings. The correlations between clinicopathological factors and DMFS were performed by univariable analysis using Log‐rank method. Variables with a *p* value <0.05 were then entered into the multivariable analysis using the Cox regression method. The survival curves were captured with the Kaplan–Meier method. *p <* 0.05 was considered statistically significant. All of the statistical analyses were performed by R software (4.15 vision) and SPSS 24.0.

## 3 RESULTS

### 3.1 Clinicopathologic characteristics and follow‐up

A total of 418 patients were included in the study, 334 patients were in the training cohort and 84 patients in the validation cohort. The baseline features between the two groups were well‐balanced. Of the 418 patients, 17.7% (*n* = 74) were stage I, 34.4% (*n* = 144) were stage II, 17.9% (*n* = 75) were stage III, and 29.9% (*n* = 125) were stage IV. More details about the tumor and treatment characteristics are available in Table 1. With the median follow‐up time of the whole cohort being 59.63 months (range 3–160 months), the estimated 3‐, 5‐, and 10‐year DMFS were 79.4%, 71.8%, and 59.1%, respectively. Moreover, the estimated 3, 5‐, and 10‐year OS were 90.2%, 81.3%, and 62.8%, respectively.

**TABLE 1:** Baseline of clinicopathological features in the two cohort

	Training *N* = 334 (%)	Validation *N* = 84 (%)	*p* value
Age	49.2 ± 14.6	50.9 ± 14.2	0.321
Gender			0.260
Male	206 (61.7)	58 (69.0)	
Female	128 (38.3)	26 (31.0)	
Tumor site			1.000
Parotid	250 (74.9)	64 (76.2)	
Submandibular	82 (24.6)	20 (23.8)	
Sublingual	2 (0.60)	0 (0.00)	
T stage			0.341
T1	94 (28.1)	25 (29.8)	
T2	155 (46.4)	45 (53.6)	
T3	39 (11.7)	5 (5.95)	
T4	46 (13.8)	9 (10.7)	
*N* stage			0.303
N0	230 (69.3)	50 (61.0)	
N1	40 (12.0)	12 (14.6)	
N2	55 (16.6)	16 (19.5)	
N3	7 (2.11)	4 (4.88)	
Pathology			0.287
High risk	76 (22.8)	14 (16.7)	
Low risk	258 (77.2)	70 (83.3)	
PNI			0.604
Negative	228 (71.9)	59 (75.6)	
Positive	89 (28.1)	19 (24.4)	
LVI			0.159
Negative	312 (93.4)	74 (88.1)	
Positive	22 (6.59)	10 (11.9)	
Surgical margin			0.323
Negative	320 (95.8)	83 (98.8)	
Positive	14 (4.19)	1 (1.19)	
Chemotherapy			0.063
No	301 (90.1)	69 (82.1)	
Yes	33 (9.88)	15 (17.9)	
PLR	132 ± 58.2	128 ± 53.5	0.615
NLR	2.20 ± 1.22	2.21 ± 0.94	0.885

Abbreviations: LVI, lymphovascular invasion; NLR, neutrophil‐to‐lymphocyte ratio; PLR, platelet‐to‐lymphocyte ratio; PNI, perineural invasion.

### 3.2 Establishment of the nomogram

In the training cohort, the continuous variables (age, NLR, and PLR) were transferred as categorical variables with the cutoff values (age = 49, NLR = 2.45, PLR = 153.1). In univariate analysis, age (*p* = 0.001), gender (*p* = 0.002), T3‐4 stage (*p* = 0.002), positive node involvement (*p* < 0.001), high‐risk pathology (*p* < 0.001), PNI (*p* < 0.001), LVI (*p* = 0.03), chemotherapy (*p* < 0.001), and PLR (*p* < 0.001) were associated with worse DMFS (Table 2). The multivariate analysis showed that age ≥ 49 (*p* = 0.033), advanced T stage (*p* = 0.003), positive *N* stage (*p* < 0.001), high‐risk pathology (*p* = 0.011), and PLR ≥153.1 (*p* = 0.033) were independent predictors for worse DMFS.

**TABLE 2:** Univariable and multivariate analysis for DMFS in the training cohort (*n* = 334)

	Univariable analysis		Multivariate analysis	
	HR (95% CI)	*p* value	HR (95% CI)	*p* value
Age		0.001		0.033
<49	Ref.		Ref.	
≥49	2.08 (1.34–3.23)		1.653 (1.040–2.63)	
Gender		0.002		
Male	Ref.			
Female	0.48 (0.3–0.77)			
T stage		0.002		0.003
T1–2	Ref.		Ref.	
T3–4	1.94 (1.27–2.97)		1.990 (1.259–3.15)	
*N* stage		<0.001		<0.001
N0	Ref.		Ref.	
N+	3.83 (2.53–5.8)		2.774 (1.747–4.40)	
Pathology		<0.001		0.011
Low‐risk	Ref.		Ref.	
High‐risk	4.35 (2.01–9.41)		2.844 (1.270–6.37)	
PNI		<0.001		0.08
Negative	Ref.		Ref.	
Positive	1.9 (1.37–2.63)		1.425 (0.959–2.12)	
LVI		0.03		0.851
Negative	Ref.		Ref.	
Positive	2.07 (1.07–4)		1.079 (0.489–2.38)	
Margin		0.805		
Negative	Ref.			
Positive	1.13 (0.42–3.1)			
Chemotherapy		<0.001		0.52
No	Ref.		Ref.	
Yes	2.79 (1.64–4.73)		1.210 (0.675–2.17)	
NLR		0.929		
<2.45	Ref.			
≥2.45	1.02 (0.65–1.6)			
PLR		<0.001		0.001
<153.1	Ref.		Ref.	
≥153.1	2.3 (1.52–3.48)		2.033 (1.323–3.12)	

Abbreviations: LVI, lymphovascular invasion; NLR, neutrophil‐to‐lymphocyte ratio; PLR, platelet‐to‐lymphocyte ratio; PNI, perineural invasion.

**FIGURE 2:**
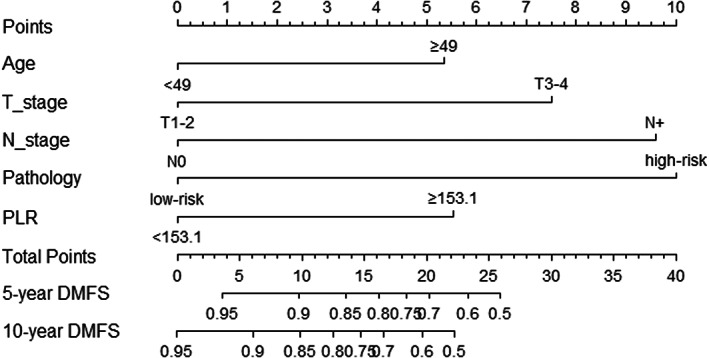
Nomogram for predicting DMFS of the major SGCs following PORT

**FIGURE 3:**
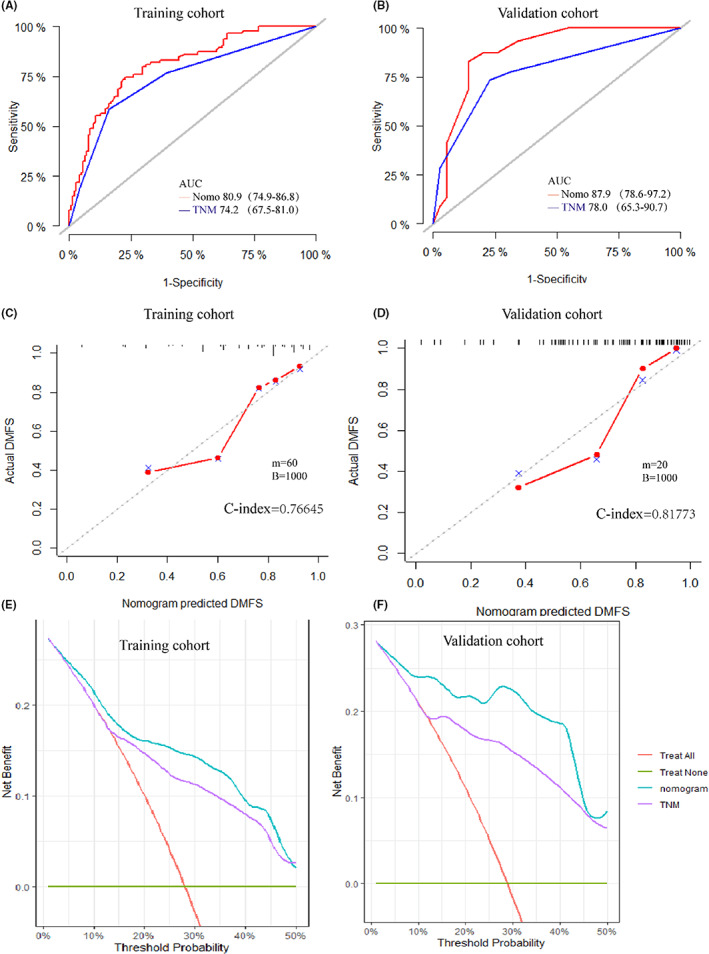
The ROC of the nomogram compared with the TNM system of the training cohort (A) and validation cohort (B) (red line: nomogram, blue line: TNM). Calibration curves in the training cohort (C) and validation cohort (D). DCA curves of the nomogram predicting DMFS compared with TNM system in the training (E) and validation set (F). The *x*‐axis is the risk threshold, and the *y*‐axis is the net benefit. The orange line represents the assumption that no patient has DM, and the green line represents the assumption that all patients have metastasis. The blue line represents the nomogram and purple line represents TNM system

We constructed the nomogram model to predict DMFS incorporating age, T stage, *N* stage, pathology, and PLR (Figure 2). The final risk score was calculated by adding up the score of each item. The C‐index of the nomogram in the training was 0.766 (Figure 3C). The AUC of the model was 80.9 (95% CI 74.9–86.8), which was greater than AUC of the TNM stage system (AUC = 74.2) (Figure 3A).

### 3.3 Validation of the nomogram

In the validation cohort, the efficacy of the model was evaluated using the calibration plot and ROC curves. The C‐index of the validation set was 0.817 (Figure 3D), which demonstrated high consistency between the predicted and actual DMFS of the model in the validation set. Additionally, the AUC of the nomogram in the validation set was 87.9 (95% CI 78.6–97.2), which showed greater ability predicting DMFS compared with the TNM system (AUC = 78, 95% CI: 65.3–90.7) (Figure 3B).

### 3.4 Clinical utility of the model

**FIGURE 4:**
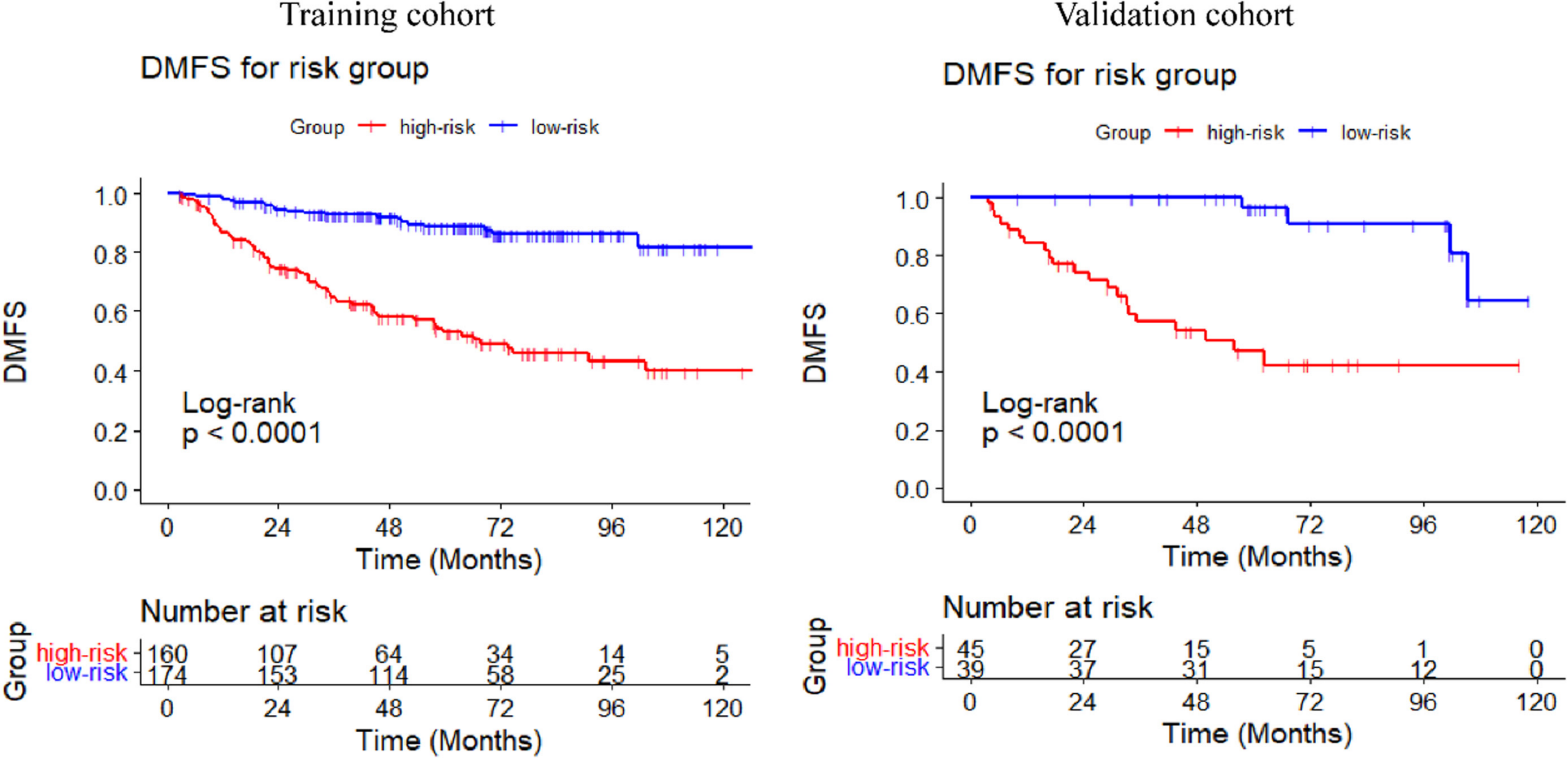
Kaplan–Meier curves of DMFS for the patients in the high‐ and low‐risk subgroups of the training and validation cohort

The DCA curves were performed for the training and validation cohort, and both indicated greater net benefit of the nomogram than the TNM system (Figure 3E,F). Additionally, the patients of the training cohort were divided into two subgroups based on median value of the total point calculated by the nomogram (Point = 15.5277). The DMFS in the high‐risk subgroup was significantly worse than that in the low‐risk group (*p* < 0.001) (Figure 4). The estimated 5‐year DMFS for the high and low‐risk subgroups were 52.8%, and 88.5%, respectively. Furthermore, risk point was calculated for every patient in the validation cohort by the nomogram of the training cohort. The patients were divided by the same the point in the training set, and significant difference was observed between the two subgroups of the validation set (*p* < 0.001) (Figure 4). The estimated 5‐year DMFS of the two groups were 46.7%, and 96.3%, respectively.

## 4 DISCUSSION

Distant metastasis appeared to be the major cancer‐related cause of death for the SGCs after PORT. In this article, we developed and validated a nomogram combing the clinicopathological features and PLR to predict the DMFS of major SGCs. The evaluation of the nomogram showed great predicting accuracy. Moreover, a risk stratification model based on the nomogram could be helpful to identify the patients presented with poor DMFS, and those whose outcomes may be improved with adjuvant therapy.

There are no randomized studies for major SGCs investigating the prognostic factors for major SGCs owing to the low incidence and diversity of pathology. Radiotherapy after surgery was considered as an important strategy to improve local control rate and OS for the cases at high risk.^20^, ^21^ Nevertheless, distant metastasis remained the critical and leading cause of death in high‐risk cases. The 5‐year distant control rate was 73%–79%,^3^, ^7^ which was consistent with the results of our study.

Given the complex diversity of histology, and the aggressiveness of pathological subtypes varied. Of which, the high‐grade histology was correlated with decreased DMFS^6^ in the SGCs. In addition, salivary duct carcinoma was one of the rare pathologies which presented with high rate of distant metastasis.^22^ Thus, we classified the histology into the high‐ and low‐risk subgroups based on the World Health Organization histological subtype. In our findings, the high‐risk histology was an independent predictor for DMFS (*p* < 0.001). Moreover, advanced T stage, *N* stage and age were significantly associated with disease‐free survival (DFS)in the SGCs.^23^ The prognostic role of lymph node involvement for OS and DMFS in the SGCs have been demonstrated in several retrospective studies.^24^, ^25^, ^26^ Additionally, several pathological features indicated predicting value for distant metastasis of major SGCs. The PNI was an independent factor associated with decreased DMFS.^27^ In our study, statistical significance was observed in the univariable analysis for PNI (*p* < 0.001) but not in the multivariate analysis (*p* = 0.08). Non‐statistical significance was observed in another study of PNI for DMFS.^28^ PNI was correlated with high‐risk pathology and advanced stages. The significant association between DMFS and PNI may be attributed by the prognostic factors of advanced stage or pathology, and independent predictive value of PNI should be reconsidered when adjusting the prognostic features.

The systemic therapies were effective approaches to improve distant control rate. However, adjuvant chemotherapy remained controversial and poorly used in patients with major SGCs with or without radiotherapy, and chemotherapy was administrated in only 11.4% of the patients in our cohort (Table 2). The host inflammatory in the microenvironment contributed to the development and metastasis^29^ of the tumors. The tumor‐infiltrating lymphocytes were associated with prognosis, and platelets were correlated with several cellular growth factors to promote tumor proliferation and progression.^30^ The elevated PLR was significantly associated with poor diagnosis in several tumors.^30^, ^31^, ^32^ Similarly, our finding demonstrated that the higher PLR (≥153.1) was an independent predictor for DMFS in the major SGCs.

Compared with the conventional prognostic model, nomogram presented with accuracy individual survival probability.^33^ J Lukovic et^34^ have built a nomogram predicting distant metastasis for the major SGCs with several clinicopathological features. However, most of the reports about the nomogram^35^, ^36^, ^37^ for the major SGCs have enrolled the patients with or without PORT owing to the rarity. PORT was significantly correlated with favorable prognosis for the major SGCs, and we have included the cases that underwent PORT. Furthermore, we have incorporated the immune‐inflammation index of PLR into the predicting model, which showed greater predictive ability than TNM system. Moreover, the nomogram indicated effective discrimination for DMFS (*p* < 0.001), and we believe that our stratification system could effectively identify the subgroup cohort at high risk of distant metastasis after PORT in the major SGCs.

To our knowledge, this study was the first to illustrate the association of immune‐inflammation index with DMFS in the major SGCs, and a nomogram was developed and validated in the cohort. Nevertheless, there are several limitations to this study. First, the inherent of the retrospective design may lead to selection bias. Second, patterns of local recurrent were not analyzed in this study. Lastly, lacking external validation makes the study more defective. Considering the rarity and histological complexity, there is clinical significance in this study. Identifying the subgroups at high risk of poor prognosis may provide a supplemental basis for adjuvant therapy in the major SGCs following PORT.

## 5 CONCLUSION

We have constructed an effective nomogram incorporating PLR with clinicopathological features and corresponding a risk stratification for predicting DMFS of the major SGCs following PORT. The nomogram showed favorable performance and risk stratification may be helpful in clinical practice and criteria of clinical trials for adjuvant therapy of the major SGCs. Further external validation is still warranted among other institutions.

## AUTHORS CONTRIBUTION

Wenbin Yan, Xiaomin Ou, Chunying Shen, and Chaosu Hu were responsible for the conceptualization, methods, analysis, writing, and editing. Wenbin Yan and Xiaomin Ou were responsible for statistical analysis and writing; Chunying Shen and Chaosu Hu were responsible for the supervision, and final editing. All authors have read and approved the final manuscript.

## CONFLICT OF INTEREST

The authors declare no conflict of interest.

## ETHICS APPROVAL STATEMENT

The study was approved by the ethics committee of the ethics committee from Fudan University Shanghai Cancer Center (SCCIRB) (2011227–11). The study did not require written informed consent for retrospective analysis.

## Data Availability

We declare that the data that support the findings of this study are available on request from the corresponding author, Chaosu Hu. The data are not publicly available due to their containing information that could compromise the privacy of research participants.
